# Melanin-Related Materials in Electrochemical Sensors for Monitoring the Environment and Food

**DOI:** 10.3390/bios15090631

**Published:** 2025-09-22

**Authors:** Agata Pane, Silvia Vicenzi, Chiara Mattioli, Dario Mordini, Arianna Menichetti, Marco Montalti

**Affiliations:** 1 Department of Chemistry “Giacomo Ciamician”, University of Bologna, Via Selmi 2, 40126 Bologna, Italy; 2 Tecnopolo di Rimini, Via Campana 71, 47921 Rimini, Italy

**Keywords:** sensing, electrochemistry, polydopamine, amplification, sensitivity, polymerization, oxidation, film, conductivity, biomedical

## Abstract

Melanin-related materials efficiently emulate the adhesion properties of natural mussel filaments and have been used advantageously for surface modification and for fabrication of electrochemical sensors for detection of environmentally relevant targets. The most applicable advantages of melanin-based coatings are their biocompatibility and versatility, and they can be easily prepared and modified according to simple and highly environmentally friendly procedures. For these reasons, melanin-related materials, in particular polydopamine, which can be obtained simply via oxidative polymerization of dopamine in an aqueous solution in the presence of atmospheric oxygen, have been applied in a large variety of scientific and technological fields. Here, we summarize and critically discuss the most recent and important applications of melanin-related materials in the development of electrochemical sensors for monitoring the environment and food. In particular, the examples used in this paper include toxic metal ions, drugs, and pesticides. In the final section of this paper, the actual limitations of the existing approach are discussed and possible future design improvements are suggested.

## 1. Introduction

Melanin is a widespread pigment found in nature. It is present in different forms [1,2] in many organisms, where it primarily serves to provide coloration [3,4] and photoprotection [5,6,7,8]. As a component of several living organisms, melanin is highly biocompatible, a property that strongly contributes to its application in fields of high social and economic impact related to human health, energy conversion, water purification, and artwork restoration [9,10,11,12,13]. In human beings, melanin is produced through a process called melanogenesis, starting with tyrosine as a molecular precursor [2]. Although the actual structure of melanin is still largely ignored [14,15,16,17] and both covalent and supramolecular [18] interactions are involved in its formation [19,20], it is known to be formed through oxidative polymerization of a precursor molecule. Due to this, a common and practical way to classify the different types of melanin is based on the specific molecular units from which they are formed [1]. Although different sources of natural melanin are available [21], e.g., cuttlefish ink, the limited versatility of natural melanin presents a relevant drawback of its application [22,23]. Modifying the morphology and the physical and chemical properties of previously formed melanin is more complicated than controlling these features during melanin formation. For this reason, different processes for producing biomimetic melanin starting from a molecular precursor, in particular dopamine (DA), have been developed [24,25,26]. This biomimetic melanin, i.e., polydopamine (PDA), is so similar to the natural type that it is considered an ideal model for eumelanin. It has also been demonstrated that PDA nanoparticles (NPs) are internalized by human keratinocytes, presenting a photoprotective action comparable to that of natural eumelanin [27].

## 2. Role of Melanin for Electrochemical Sensors

**Figure 1:**
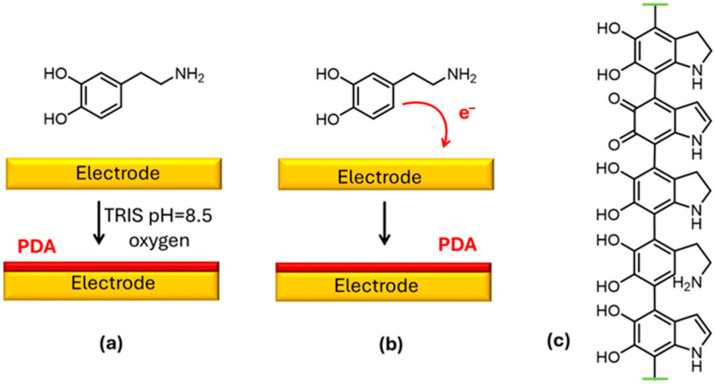
(**a**) Chemical deposition of a PDA film on an electrode. (**b**) Electrochemical deposition of a PDA film on an electrode. (**c**) A possible chemical structure of PDA.

Melanin-like materials, in particular PDA, present a peculiar combination of features that make them very useful in the design of electrochemical sensors for environmental applications. First of all, these materials are highly biocompatible and can be prepared under very mild conditions [22]. Messersmith and his coworkers demonstrated that any kind of material can be coated with PDA simply by starting from DA in water, under controlled pH conditions, using a Tris buffer (Figure 1) [25]. The adhesion of PDA films to substrates relies on surface interactions similar to those used by mussels, primarily involving the catechol and amino groups of dopamine. Indeed, PDA presents a high density of functional groups that can be involved in several kinds of chemical reactions and interactions [28]. Hence, PDA coating not only guarantees efficient adhesion to any type of surface, but it also allows a high functionalization density to be achieved [25]. A further advantage of PDA in the preparation of electrochemical sensors is that, as schematized in Figure 1, it can be prepared directly by electrochemical deposition [29].

The electrical conductivity of melanin depends on its hydration state, progressing from moderate to low, i.e., 10^−5^–10^−13^ S cm^−1^ [30,31]. Studies of Sepia melanin [32] (extracted from cuttlefish ink) revealed that conductivity can be increased to 10^−3^ S cm^−1^, an outstanding value for natural organic materials [33]. Pioneering studies on both natural and biomimetic melanin, on the other hand, reveal that melanin’s electrical conductivity undergoes an off–on transition when an increase in voltage is applied [30]. The transition voltage decreases with reduced sample thickness, reaching approximately 10 V for a 0.1 mm sample, while the post-transition conductivity increases by 2–3 orders of magnitude [30]. These results suggest that applying electrical conductivity data obtained for bulk melanin-related materials can be inappropriate at the nano scale, since transition voltages, which produce large increases in conductivity, are expected to be very low for ultrathin films. Moreover, the conductivity of a melanin film is determined by the mechanism of conduction, which can be very different in various films [32]. In this context, it has been demonstrated that highly conductive PDA films can be obtained by direct electrochemical synthesis on gold electrodes [31]. Nevertheless, we stress that melanin-related materials are not considered good conductors. For this reason, in most of the examples we discuss in this review paper, they are used for preparation of electrochemical sensors in combination with other nanomaterials with higher electrical conductivity, like graphene or gold NPs. Methods to increase the conductivity of melanin and, in particular, PDA include graphitization [34], metal doping [35], and hybridization [36,37]. For example, after pyrolysis, the electrical conductivity of PDA was reported to be as high as 1.2 × 10^5^ S m^−1^ [38]. We stress that, while incorporation of melanin in electrochemical sensors is recognized as an efficient strategy to increase interactions with the target analyte and hence the sensitivity, the actual effect of the production process on the conductivity of melanin itself is largely ignored. By discussing the most representative examples of application of melanin-related materials reported during the last five years, we will demonstrate that incorporation of these materials leads to increases in the performance of electrochemical sensors for the detection of environmentally relevant species. For some applications, changes in the photochemical and optical properties are considered together with changes in the electrochemical ones. Melanin-based optical sensors were discussed in detail in [39]. In the final section, we will outline possible perspectives and developments for this analytical strategy.

### 2.1. Melanin Formation and Functional Groups

Melanin formation is, in general, an oxidative polymerization process. One natural precursor of melanin is L-tyrosine, which is enzymatically oxidized in the presence of oxygen to form eumelanin [40]. This process leads to the formation of a catechol (L-DOPA), which is further oxidized to quinone. Catechol derivatives, in general, produce quinones upon oxidation, which quickly react with the catechol molecules themselves [18]. This mechanism is quite general, and it can be extended to different precursors. The main difference between the precursors is their redox potentials, and milder or stronger oxidizers may be needed for polymerization [41]. In the case of electrochemical sensors, polymerization can be achieved electrochemically [29]. As discussed in detail in [28], several groups, including amines and carboxylates, are present in melanin, and they can bind effectively to metal ions [42] or interact with organic molecules [43,44].

### 2.2. Kinds of Electrochemical Sensors

Electrochemical sensors may be based on different working principles, which were recently summarized in [45] as follows:

Potentiometric sensors: They measure the electromotive force (EMF) or potential difference between a reference and a working electrode (WE), and they work with negligible current flow.

Amperometric sensors: They allow quantification of the concentrations of target electroactive species by measuring the current generated through their oxidation or reduction at an electrode under a constant applied potential.

Voltammetric sensors: They analyze current responses with respect to a systematically varied applied potential. Depending on the kind of potential profile, they are classified as cyclic voltammetry (CV), stripping voltammetry (SV), square-wave voltammetry (SWV), linear sweep voltammetry (LSV), differential pulse voltammetry (DPV), etc. Advantages and disadvantages of the different methods are listed in [45]. Other kinds of electrochemical sensors are conductometric [46], impedimetric [47], and coulometric [45].

## 3. Environmental Applications

Before the Industrial Revolution, natural pathogens like bacteria and viruses were the primary environmental threats to health and ecosystems. After industrialization, pollution dramatically shifted, introducing new contaminants such as heavy metals and industrial chemicals into the environment [48].

Nowadays, pollutants in the environment represent a major global issue that has large impacts on both ecosystems and the human body. Pollutants are defined as any substance or form of energy introduced into the environment that has undesirable effects on its surroundings. Some of them are naturally occurring, such as biological toxins, while others are released into atmospheric, aquatic, and terrestrial systems by contemporary societies through industrial outputs, agricultural practices, and domestic waste generation [49].

To address this problem, several chemical methods have been developed to constantly monitor and measure pollutant concentrations in environmental ecosystems. Exploiting electrochemical methods for pollutant detection, such as with electrochemical sensors, gives on-site monitoring, achieving high sensitivity and selectivity.

### 3.1. Heavy Metal Ion Detection

Toxic heavy metals are mainly released by industries as by-products, and they can contaminate the environment, leading to significant health risks and ecosystem damage. Heavy metal ions (HMIs), such as lead (Pb), cadmium (Cd), copper (Cu), nickel (Ni), chromium (Cr), and arsenic (As), with densities higher than 5 g/cm^3^ are toxic and non-biodegradable. The main health concerns related to HMIs are damage to the central nervous system; cardiovascular and gastrointestinal disorders; and diseases affecting the lungs, kidneys, and liver [50]. Traditional methodologies to detect these contaminants rely on analytical techniques, such as High-Performance Liquid Chromatography (HPLC), Mass Spectrometry (MS), and capillary electrophoresis. However, these techniques are expensive and difficult to use. For this reason, new methodologies involving electrochemical approaches are being studied and developed. The examples discussed in this subsection are summarized in Table 1 and Table 2.

The performance of sensing electrodes for detection of HMIs is strictly connected to the material from which they are constructed. Traditionally, graphene materials have been widely exploited due to their many advantages. Patel et al. presented the development of an alanine-decorated polydopamine-coated reduced graphene oxide (ALA/PDA/rGO) nanocomposite for simultaneous electrochemical detection of Cd^2+^, Pb^2+^, Cu^2+^, and Fe^2+^ ions in solution. The choice to use graphene as an electrode material was made due to its high electrical conductivity, large surface area with many active sites, and strong electrocatalytic properties. However, simple graphene lacks an affinity for adsorbing metal ions, making it unsuitable for HMI detection. Therefore, the authors synthesized and used a derivative, reduced graphene oxide (rGO), which is characterized by a large surface area and high electrocatalytic and electrical conductivity. To overcome the problem of self-aggregation of rGO, which limits its performance, PDA was used. PDA acts as a reducing agent for graphene and a stabilizing agent for rGO. Due to its high contents of functional groups, it can provide a large number of active sites to enhance metal chelation. Alanine was included to further increase the metal-binding capabilities of the system. DPV was used to assess different electrochemical parameters such as the potential and the metal deposition time. Metal ions were successfully detected by the sensor with high sensitivity. The sensor was also tested successfully in real water samples containing HMIs, and it showed low interference, high stability, and reproducibility [51].

**Figure 2:**
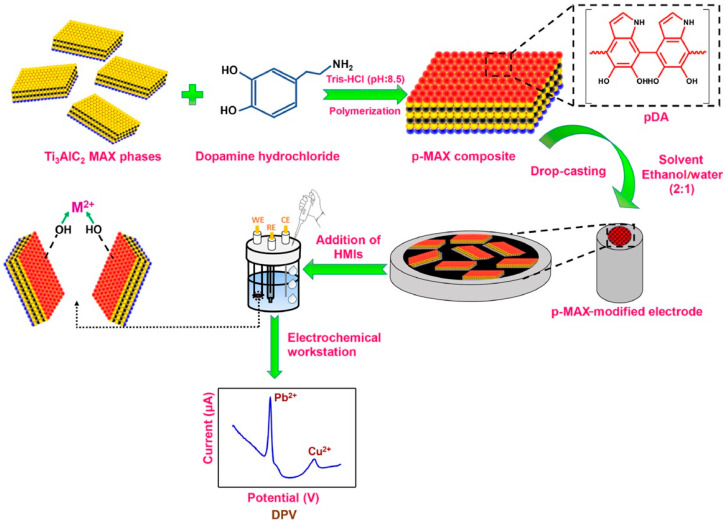
A schematic representation of the fabrication of a p-MAX-modified electrode and its application as a working electrode for detecting HMIs [50].

Besides graphene, many other materials have been investigated for the development of sensing electrodes. Some of them are MXenes. They belong to a family of 2D materials derived from selective removal of the A phases from MAX phases, resulting in ternary layered ceramic materials that are highly electrically and thermally conductive and resistant to strong conditions. MXenes are perfect candidates for metal detection due to their layered structure, high conductivity, and the ease with which their chemistry can be tuned without changing their structure. However, the process of removing the A phase from MAX phases is long and intensive and requires high precision. To address these limitations, Nema and her colleagues constructed, as schematized in Figure 2, an electrochemical sensing platform composed of a Ti_3_AlC_2_ MAX phase and mussel-inspired PDA for HMI detection, in particular Cd^2+^, Pb^2+^, and Cu^2+^. The authors focused on the less explored MAX-phase materials, avoiding the etching of the A-phase step and making the process much smoother, aiming to obtain better performance with respect to MXenes. The system was enriched with PDA, which was applied on glassy carbon electrodes as a modifying agent to increase the binding properties and the active sites available for the target metal ions. The electrochemical performance was evaluated by CV, confirming the positive sensitivity of the sensor towards the specific metal ions. In addition, some experiments to investigate the anti-interference ability of the sensor have been performed. The sensor showed positive detection of the target ions in the presence of interference ions. However, in the case of interfering agents such as Zn^2+^ and Ni^2+^, the current values for Cu^2+^ decreased, possibly due to formation of intermetallic compounds. Overall, the sensor successfully detected the target metal ions but lacked high selectivity [50].

Metal–Organic Frameworks (MOFs) are another class of material that has been widely investigated for electrochemical analysis. They are a class of porous and crystalline materials derived from coordinated bonding between metal ions and organic linkers. Their main advantages for the detection of specific target molecules are their high porosities, large surface areas, and tunable structures, which contribute to changes in their chemical and electrical properties that depend on the guest molecule they are adsorbing. A very popular MOF is Zeolitic Imidazolate Framework-8 (ZIF-8), which is composed of zinc ions linked by 2-methylimidazolate ligands. It has been employed by Zhao and his coworkers to develop ZIF-8-derived hollow mesoporous carbon cubes loaded with a Au nanoparticle (Au@HMCC) nanocomposite, which was further modified on screen-printed carbon electrodes (SPCEs) for electrochemical detection of Cr(VI). However, MOFs often lack adsorbability and stability when in an aqueous solution, and they show low conductivity. To expand the application of MOFs for electrocatalysis, the authors modified them with carbonaceous materials, which show high conductivity and catalytic activity, to obtain a product with higher porosity and a larger surface area that was able to expose more active sites for the redox processes. AuNPs were included because they possess unique hollow cavities and a low density, in addition to many internal voids. These features can increase the electrode–electrolyte contact area during electrochemical reactions. PDA was coated on the material to exert its potential, which was provided by the high number of active sites. Through catalytic Cr(VI) reduction at the electrode, it was possible to detect its concentration. Excellent results have also been obtained regarding the stability, accuracy, and reliability of this method, proving that Au@HMCCs/SPCEs can be a promising platform for detection of Cr(VI) for different applications [52].

PDA was further included in other sensing electrodes. Wang et al. synthesized Fe_3_O_4_@PDA@MnO_2_ core–shell magnetic nanocomposites for the development of an electrochemical sensor for detection of Pb(II) in environmental samples. The core of the system was a magnetic nanocomposite made of magnetic nanoparticles, Fe_3_O_4_, coated with an inorganic material, MnO_2_, and an organic material, PDA. This combination led to synergistic properties superior to those of the individual components. In fact, MnO_2_ is inexpensive and presents high stability, strong adsorption properties, and a large surface area. However, it tends to agglomerate when used on its own. PDA is a perfect polymer for protecting the magnetic core of the system due to its environmental stability, nontoxicity, and easy preparation. All the coating processes were performed under environmental conditions, which is important for practical applications. Due to the magnetic properties of the prepared sensor, Pb(II) could be easily collected and isolated from a solution and adsorbed in the coating materials, resulting in high sensitivity and selectivity for its detection and quantification [53].

**Figure 3:**
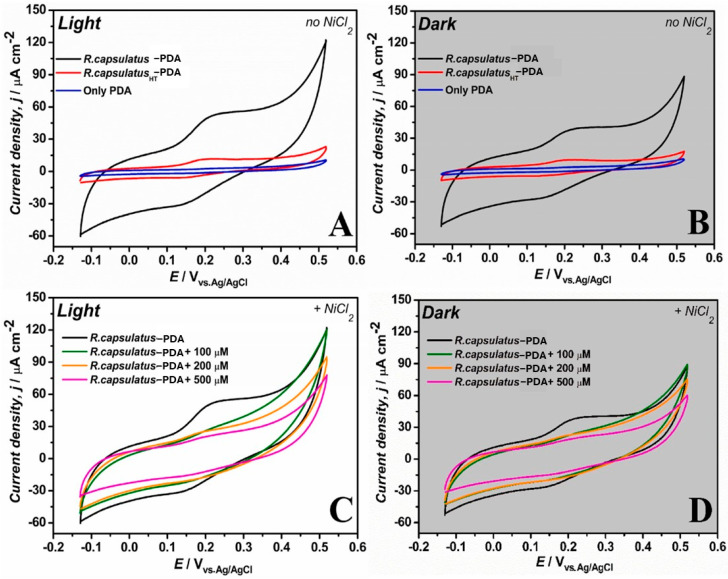
CV for the biophotoanode under light (**A**) and dark conditions (**B**) for R.caps-PDA (black line); heat-treated R.capsHT-PDA (red line); and only PDA (blue line). The performance of the R.caps-PDA biohybrid photoanodes (black line) was evaluated with exposure to 100 μM (green line), 200 μM (orange line), and 500 μM (magenta line) NiCl2 in the presence (**C**) and absence of light (**D**). CE: Pt; RE: Ag|AgCl (3 M NaCl). Scan rate = 5 mV s^−1^; temperature = 25 °C [54].

The last study that is worth mentioning for HMI detection was presented by Franco and his colleagues. They developed a unique self-powered bio-based microbial electrochemical sensor for detection of NiCl_2_ and CuSO_4_ (Figure 3). Their research is interesting because they focused on a different technique exploiting solar radiation and the ability of some types of bacteria to convert sunlight into energy. To prepare the system, the authors relied on purple non-sulfur photosynthetic bacterium (PNSB) cells, specifically Rhodobacter Capsulatus (R. capsulatus), due to their versatility, which allowed the use of different substrates and organic compounds, and due to their efficiency in converting energy from sunlight into photoexcited electrons. The electrode was made of poly-3-hydroxypolybutyrate (PHB)–carbon nanofibers (CFs) and was able to enhance the interactions with the bacteria and the conductivity of the system. Also, the bacteria were electropolymerized with a redox-adhesive PDA matrix-based coating that was able to entrap them. The principle of the working system is photobioelectrocatalysis: energy from sunlight is absorbed by the bacteria and transformed into energy to excite electrons and generate a current at the electrode surface. When in the presence of metal ions to be detected, their binding decreases the efficiency with which electrons are transferred from the bacteria to the electrode, decreasing the measured photocurrent. This decrease is correlated with the metal ion concentration; thus, it is possible to detect the presence of metal ions and their quantity in a sample. The described biosensor opened the door for future development of low-cost and self-powered biosensors able to perform in situ monitoring of heavy metals in aqueous environments [54].

Overall, electrochemical techniques offer a powerful method for sensitive and specific detection of HMIs, often exploiting the electrode surface’s ability to be modified with several materials, including PDA, which has excellent adhesion properties and is rich in functional groups that can bind different analytes, making it a promising ally for environmental monitoring.

**Table 1:** A summary of the analytical parameters discussed in papers presented in Section 3.1.

Ref.	Analyte	Analyte Concentration in Real Samples	LOD	Stability	Linear Response Range	Regulatory Threshold in Potable Water	Regulatory Threshold in Superficial Water
[51]	Cd^2+^, Pb^2+^, Cu^2+^, and Fe^2+^ ions in water and soil	50–200 ppb Spiked addition	1.46, 2.86, 17.95, 50.23 ppb, respectively	After 1 year, the electrode still detected all four HMIs.	Cd^2+^: 3–10 ppb, Pb^2+^: 9–21 ppb, Cu^2+^: 20–50 ppb, and Fe^2+^: 50–100 ppb	WHO: Cd^2+^: 0.003 mg/L, Pb^2+^: 0.01 mg/L, Cu^2+^: 2 mg/L Bureau of Indian Standards: Fe^2+^: 0.3 mg/L	WHO: Cd^2+^: 0.005 ppm, Pb^2+^: 0.05 ppm ppb, Cu^2+^: 1.5 ppm WFD: Fe2+: 0.73 mg/L
[50]	Cd^+2^, Pb^+2^, and Cu^+2^ in tap water	50–300 ppb Spiked addition	1.43, 2.41, and 2.48 ppb, respectively, in individual detection; 3.43 for Pb^2+^ and 3.47 for Cu^2+^ for simultaneous detection	Nd	Cd^+2^: 2–16 ppb, Pb^+2^: 3–15 ppb, and Cu^+2^: 8–18 ppb	WHO: Cd^2+^: 3 ppb, Pb^2+^: 10 ppb, Cu^2+^: 2000 ppb	WHO: Cd^2+^: 0.005 ppm, Pb^2+^: 0.05 ppm, Cu^2+^: 1.5 ppm
[52]	Hexavalent chromium in tap water and river water	Tap water: 0.0490; 0.506; 1.01; 4.96 μg/mL	3.0 μg L^−1^	After 30 days, the signals remained at 97.73% of their initial value.	0.01 μg mL^−1^ to 112 μg mL^−1^	EPA: 100 ppb	EPA: 18 µg/L as a 24 h average for saltwater
		River water: 0.0510; 0.515; 1.02; 4.88 μg/mL					
		Spiked additions: 0.050; 0.50; 1.0; 5.0 μg/mL, respectively					
[53]	Pb(II) in lake water	Yangzong lake water: BDL; 0.98; 10.06; 20.05 μg/L	0.03 μg L^−1^	A 3.4% decrease in the peak current after four weeks	0.1–150 μg L^−1^	WHO: Pb^2+^: 10 ppb	WHO: Pb^2+^: 0.05 ppm
		Datun lake water: 5.81; 6.78; 11.90; 15.84					
		Spiked additions: 0.00; 1.00; 10.00; 20.00 μg/L for Yangzong lake water and 0.00; 1.00; 6.00; 10.00 μg/L for Datun lake water, respectively					
[54].	NiCl_2_ and CuSO_4_ in beer	100 μM, 200 μM, and 500 μM (spiked addition)	ND	ND	ND	WHO: 70 µg/L for Ni EPA: 1.3 mg/L for Cu	ND

**Table 2:** A summary of the structural features of systems discussed in papers present in Section 3.1.

Reference	Base Electrode	Electrode Modifier	Precursors	Mechanism of Modification
[51]	GCE	DA, GO, L-Alanine	ALA/pDA/rGO	Drop casting
[50]	GCE	DA, MAX phase (Ti_3_AlC_2_)	Ti_3_AlC_2_ MAX phase, PDA	Drop casting
[52]	Screen-printed carbon electrodes	DA, ZIF-8 cubic nanocrystals, HAuCl_4_	ZIF-8, AuNPs, PDA	Drop casting
[53]	mGCE	Fe_3_O_4_ magnetic NPs, DA, KMNO4	Fe_3_O_4_@PDA@MnO_2_	ND
[54]	poly-3-hydroxypolybutyrate (PHB)–carbon nanofibers (CFs)	DA, *R. capsulatus* cells	Purple non-sulfur bacteria, redox-adhesive PDA matrix	Drop casting

### 3.2. Detecting Drugs and Pharmaceutical Products

Besides microbial pollutants, heavy metals, and priority pollutants, in recent years Emerging Pollutants (ECs), including therapeutic products, hormones, and drugs, have gained increasing attention. They can be either synthetic or naturally occurring, and they have only recently been identified as potentially hazardous to human health and ecosystems [48]. The examples discussed in this subsection are summarized in Table 3 and Table 4.

In particular, they can be responsible for bacterial resistance and toxicity toward organisms due to their persistence and accumulation in the biological chain. A key part of this problem is their modification through biological systems. Many of these molecules are volatile, and they can be degraded or metabolized, forming more persistent sulfonate and carboxylic acid versions [55].

Among the different pharmaceutical compounds that can accumulate in the environment, paracetamol (acetaminophen), a commonly used analgesic and antipyretic agent, is one of the most frequently detected substances due to its worldwide overconsumption.

When absorbed by the body, it can accumulate, causing oxidative stress, which can lead to different diseases. Even small quantities of this molecule can harm aquatic life by disrupting ecosystems and accumulating in organisms. In fact, paracetamol has been found in different water sources, including drinking water, seawater, and wastewater. Mahalakshmi and her colleagues studied a new electrochemical approach by developing a neodymium-decorated polydopamine-reduced graphene oxide (Nd-PDA-rGO) nanocomposite for ultrasensitive detection of paracetamol. Reduced graphene oxide (rGO) is an excellent candidate for sensor development due to its unique electrical, mechanical, and chemical properties. It was employed to construct the system because it has a large surface area and high conductivity. rGO was further modified to increase the number of active sites able to bind target molecules. In particular, the authors used dopamine, which can polymerize on GO, forming PDA, and improve the stability and hydrophilicity of the system. Moreover, Nd was added to further boost the sensitivity and selectivity of the sensor by adding active sites and increasing the electronic properties. By DPV, it was possible to assess that, by binding with paracetamol, the current response of the modified electrode exhibited a linear increase with an increase in the paracetamol concentration, making it possible to obtain a quantitative measurement of the paracetamol concentration. The results showed that the Nd-PDA-rGO nanocomposite exhibited excellent sensing capabilities, positively contributing to the development of advanced pharmaceutical monitoring tools [56].

Detection of paracetamol was also assessed by Tlili et al. They presented the creation of an electrochemical sensor using molecularly imprinted polymers (MIPs). These synthetic polymers are designed to mimic the highly specific recognition capabilities of natural biological receptors. By the imprinting technique, it is possible to create cavities within a polymer matrix that are complementary to the target molecules to be detected. The authors used density functional theory (DFT) to choose the best polymer to coat the electrode and obtain the highest sensitivity and specificity towards paracetamol. Through analysis of the electronic structure and reactivity of a monomer, this technique is able to predict which molecule best fits the desired objective. Dopamine was selected as the best MIP, as it exhibited the lowest interaction energy in the liquid phase. Dopamine was electropolymerized in the presence of paracetamol to obtain a selective template for this molecule. Through SWV, it was possible to measure the current peak modifications, which were correlated to paracetamol’s binding to the electrode. The developed electrochemical sensor showed excellent performance and a wide detection range with a low detection limit and high sensitivity. Its selectivity was demonstrated using common interfering agents like tyrosine, proline, 4-nitrophenol, and vitamin B3. The sensor successfully identified paracetamol in real-world samples, suggesting practical reliability [57].

**Figure 4:**
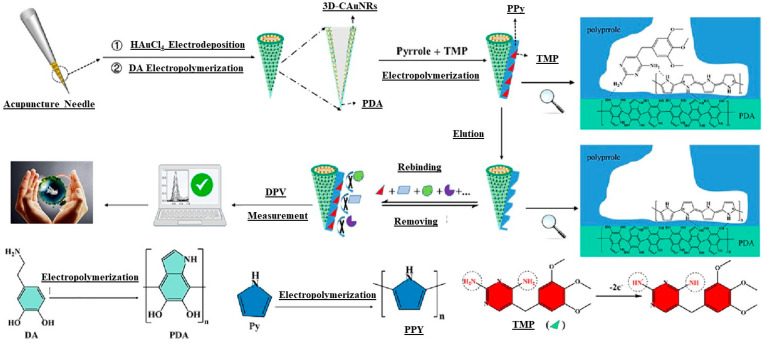
A schematic illustration of the fabrication procedure for MIP/pDA/3D-CAuNRs/ANME and the procedures for recognizing and measuring TMP [58].

Other studies also employed MIPs to exert their chemical properties and advantages. In particular, Liu et al. presented a novel method for detecting Trimethoprim (TMP), a highly efficient antibacterial agent that can be found as a residue in the environment, affecting organisms and increasing bacterial resistance. They used acupuncture, an efficient method for treating diverse diseases that comes from traditional Chinese medicine. As schematized in Figure 4, acupuncture needles are made of stainless steel, which shows many advantages compared to other electrode materials such as gold, including its cheapness and excellent electrical conductivity. The authors developed a novel micro-detector that can be added to the matrix of an acupuncture needle for detection of TMP. They focused on synthesizing MIPs by a green electrosynthesis route by incorporating three-dimensional coral-like gold nanorods (3D-CAuNRs), PDA, polypyrole (pPY), and TMP, which were electropolymerized onto the acupuncture needle. The resulting system exhibited excellent selectivity for detecting TMP in environmental water and soil, showing a wide linear range and a low limit of detection for TMP [58].

Bacterial resistance is a real and serious issue. It is a natural process where bacteria evolve, by genetic changes, to escape the effects of drugs that kill them, such as antibiotics. In addition to TMP, other antibacterial agents can accumulate in the environment. Song and her coworkers developed an aptasensor (Au@PDA@NH2-MIL-101(Fe)), a type of biosensor that uses aptamers as biorecognition elements, for detection of Oxytetracycline (OTC), an antimicrobial drug. Aptamers are single-stranded nucleic acid or peptide molecules that are able to bind to specific target molecules. They have been integrated into sensors due to their high affinity, specificity, and stability. This particular electrochemical sensor was innovatively built by combining AuNPs modified with self-polymerized dopamine and iron-based metal–organic frameworks (Fe-MOF). AuNPs were chosen for their excellent conductivity and biocompatibility, while dopamine was employed for its strong adhesion, biocompatibility, and signal amplification capabilities. Also, its self-polymerization process on the AuNPs’ surfaces guaranteed strong interactions with biomolecules. Fe-MOFs served as active sites for aptamers, enhancing the functionality. All the features enhanced the electrochemical signal and the sensitivity for OTC. When in the presence of OTC, aptamers could positively identify it and bind to it. By DVP, it was possible to observe that, when bound, electron transfer was blocked on the surface of the electrode, decreasing the current. This decrease was proportional to the increase in the concentration of OTC in the sample [59].

Another investigated class of pharmaceutical compounds includes antipsychotic drugs. Chlorpromazine hydrochloride (CPZ) is a widely prescribed first-generation antipsychotic employed in the management of schizophrenia, bipolar disorder, and severe behavioral disorders. Nevertheless, its protracted therapeutic use is linked to a range of adverse effects, including but not limited to blurred vision, movement disorders, sexual dysfunction, and altered consciousness. Furthermore, environmental dissemination of CPZ into surface waters has demonstrated ecotoxicological impacts on aquatic ecosystems, thereby necessitating enhanced efforts to achieve accurate environmental detection. Two different research groups focused their studies primarily on developing MIP-based electrochemical sensors (ECSs) for CPZ quantification. Both approaches leverage PDA and carbon-based materials to develop highly sensitive, selective, and stable sensors for CPZ, demonstrating excellent performance when analyzing real samples and exhibiting low detection limits and wide linear ranges. However, their specific design strategies differ: Yuan et al. developed a novel ratiometric electrochemical sensor based on PDA and ZnMn_2_O_4_/carbon nanotubes (ZMO/MWCNTs), as it both enhanced electron transfer and offered ample surface area for loading the MIP. The MIP film was then electropolymerized onto the GCE, using DA coordinated with copper ions as the monomer. This PDA MIP served dual purposes: acting as the molecular imprint while also functioning as an internal reference to boost measurement reliability by correcting for fluctuating conditions. Adding copper ions further improved imprinting efficiency and lowered charge transfer resistance. This Cu-MIP/ZMO/MWCNTs/GCE sensor enabled CPZ quantification over a wide linear range due to electrochemical oxidation of CPZ after selective binding to MIP sites [60].

In contrast, Zhou et al. focused on an ultrasensitive MIP/ECS built using nitrogen-doped hollow mesoporous carbon spheres (N-HCSs), employing DA and 3,4-ethylenedioxythiophene (EDOT) as dual-functional monomers that polymerize to create an imprinted network capable of highly selectively recognizing CPZ and form a donor–acceptor electron system that promotes electron transfer. Their study illustrates progressive enhancement of the response current with rising concentrations of CPZ. This increase is attributed to the superior affinity of the imprinted cavities for CPZ, the efficient donor–acceptor electron system of PDA with PEDOT, and the robust electron transfer capabilities of NHCS. Employing the background-subtracted DPV method, a linear correlation was established between the concentration of CPZ and the response current values of its oxidation peak [61].

While both of these research articles introduce a promising approach for constructing electrochemical sensors with enhanced selectivity, sensitivity, and stability, the study by Zhou et al. reports a slightly lower limit of detection and a broader detection range.

Besides pharmaceutical drugs, ECs can also include molecules generated by organisms themselves. An example is β-Nicotinamide adenine dinucleotide (NADH). It is a form of vitamin B3, and it is essential for cellular viability. It is continuously synthesized by our bodies but can also be absorbed through supplements and food, leading to overdoses of this molecule in our bodies. In fact, excessive levels of NADH could be related to neurological diseases, diabetes, and cancer, giving its detection fundamental importance. Prasanna et al. developed a composite containing polydopamine and titanium carbide with gold nanoparticles (Au@PDA/TiC) for the detection of NADH in food and environmental samples. The authors chose to employ titanium carbide (TiC) for the construction of the composite. This molecule shows high efficiency for sensing due to its stability and electrocatalytic features. However, TiC can agglomerate, limiting its surface area. To address this limit, the authors added a PDA coating on the sensor, enhancing its sensitivity thanks to a larger surface area and strong adhesion. AuNPs were included to increase sensor conductivity and biocompatibility by amplifying the signal. The composite enabled NADH oxidation at a very low potential, demonstrating significant potential for advanced sensing applications [62].

All these studies highlighted the highly promising approach of electrochemical sensing for on-site monitoring of pharmaceutical contaminants in the environment, safeguarding ecological systems and public health.

**Table 3:** A summary of the analytical parameters discussed in papers presented in Section 3.2.

Ref	Analyte	Analyte Concentration in Real Samples	LOD	Stability	Linear Response Range	Regulatory Threshold in Potable Water	Regulatory Threshold in Superficial Water
[56]	PA	ND	0.1 μM	After 2 days, 91.17% of the original redox peak current	from 0.3 to 10 μM	ND	ND
[57]	PA in seawater, hospital effluent, and wastewater	10^−10^ mg/mL to 3.3 mg/mL (spiked addition)	0.55 × 10^−10^ mg/mL	ND	ND	ND	ND
[58]	TMP in soil and lake water samples	Soil: 18.33; 29.75; 37.19; 48.69 μmol/L	0.017 μmol/L	The signal response decreased to 95.3% of the original current response after 7 days.	0.05–50 μmol/L	ND	ND
		Lake water: 19.75; 29.60; 39.44; 47.35 μmol/L					
		Spiked additions: 20.00; 30.00; 40.00; 50.00 μmol/L, respectively					
[59]	OTC in tap water samples	With DPV: ND; 0.97; 9.82 μM	6.88 nM with DPV and 5.56 nM with CV	The oxidation current signal of OTC retained 87% of its initial value after 30 days.	10 nM–10^4^ nM	ND	ND
		With CV: ND; 1.02; 9.94 μM					
		Spiked additions: 0; 1; 10 μM, respectively					
[60]	CPZ in human serum, urine, and lake water samples	Human serum: 56.0; 92.0; 472 nM	0.42 nM	After 14 days, the oxidation peak currents of PDA and CPZ retained 95.2% and 96.5% of their initial values, respectively.	1 nM–10 μM	ND	ND
		Urine: 53.0; 105.0; 483 nM					
		Lake water: 53.0; 91.0; 465 nM					
		Spiked additions: 50.0; 100; 500 nM, respectively					
[61]	CPZ in milk, eggs, and lake water	Milk: 0.00493; 0.0995; 0.505 μM	0.18 nM	After 4 weeks, the peak current response maintained 95.9% of its initial value.	0.0005–85 μM	ND	ND
		Eggs: 0.00518; 0.103; 0.497 μM					
		Lake water: 0.00517; 0.0988; 0.505 μM					
[62]	β-NADH in food and environmental and biological samples	Avocado juice, apple juice, human serum, human urine, lake water, and river water samples Spiked addition using amperometric technique: 0–30 µM DPV: 0–25 µM	amperometric technique: 0.0062 μM DPV: 3.17 μM	After 15 days, the current intensity retained 73.6% of its initial value.	Amperometric technique: 0.018–674 μM DPV: 5–450 μM	ND	ND

**Table 4:** A summary of the structural features of the systems discussed in papers presented in Section 3.2.

Reference	Base Electrode	Electrode Modifier	Precursors	Mechanism of Modification
[56]	GCE	Neodymium nitrate hexahydrate (Ni), DA, graphite powder	Nd/PDA-rGO	Drop coating
[57]	Gold electrode	DA	MIP (PDA)	Electropolymerization
[58]	ANME	3D-CAuNRs, PDA, pPY, TMP	MIP/pDA/3D-CAuNRs/ANME	Chronoamperometry scanning, electropolymerization
[59]	GCE	DA, AuNPs, FeCl_3_⋅6H_2_O, EDC/NHS	Apt/Au@PDA@NH_2_-MIL-101(Fe)	Coating (not specified)
[60]	GCE sensor	Cu-MIP/ZMO/MWCNTs	Zn (AC)_2_⋅2H_2_O; Mn(AC)_2_⋅4H_2_O; urea; MWCNTs; tetrabutylammonium tetrafluoroborate (TABTFB); dopamine; CuCl_2_⋅2H_2_O	Drop coating, electropolymerization
[61]	GCE sensor	N-HCS@MIP	Tetraethoxysilane; (3-aminopropyl)triethoxysilane; dopamine hydrochloride; silica nanoparticles; N2; EDOT;	Drop coating
[62]	GCE	DA, HAuCl4, bulk TiC powder	Au@PDA/TiC	Drop casting

### 3.3. Detecting Pesticides and Other Related Contaminants

Pesticides are widely utilized globally to protect crops, aiming to optimize agricultural yields. However, their application, if not managed carefully, can affect organisms beyond the intended pests, including humans. Research indicates that exposure to these compounds has been associated with various health effects in humans, such as endocrine system alterations, immune system responses, reproductive health challenges, and cellular toxicity. This presents significant conflict between agricultural productivity and potential health impacts [63]. The examples discussed in this subsection are summarized in Table 5 and Table 6.

Current lab methods are too slow for rapid, accurate monitoring, highlighting an urgent need for portable, accurate on-site detection. Electrochemical sensors are gaining traction for this, offering simplicity, low costs, and fast analysis, making them ideal candidates for real-time environmental monitoring [64].

Driving this advancement in pesticide detection, a paper by Luo et al. introduced a new electrochemical sensor specifically designed to meet the need for portable and accurate tools to detect organophosphorus pesticide residues, particularly focusing on detection of dichlorvos in farmland water. Dichlorvos, an organophosphorus pesticide, is currently in use and is categorized as an EC. The researchers developed a sensor using a novel material called ZrO2@PDA combined with a glassy carbon electrode (GCE) and tested its performance. The sensor detects dichlorvos by measuring changes in electrical signals that occur when dichlorvos interacts with the ZrO2@PDA-modified electrode’s surface. The ZrO_2_ nanofilm enhanced electron transfer and catalytic activity, while PDA, with its open mesoporous channels, improved analyte contact and signal strength while also helping to immobilize the sensing materials on the GCE. The sensor demonstrated good reproducibility, stability, selectivity, and anti-interference capabilities against common ions and organic substances present in water bodies, offering a promising alternative to traditional, resource-intensive laboratory methods [64].

Chen et al. further illustrated progress in current pesticide monitoring, focusing on Atrazine (ATZ). Among widely used herbicides, ATZ is considered effective and inexpensive, but it is persistent, and its presence in the environment can affect human health. In this context, the development of sensitive and selective detection methods has become crucial. They developed a photoelectrochemical (PEC) aptamer sensor for detection of ATZ based on cascade signal amplification. The biosensor was synthesized utilizing a Cd0.5 Zn0.5S/Ti_3_C_2_ photoelectric material and was then immobilized on a fluorine-doped Tin Oxide (FTO) electrode as a photoanode. On the electrode’s surface, the G-quadruplex (G4)/hemin DNAzyme was deposited. The sensor’s operational principle involves determination of ATZ, leveraging signal amplification through the cascade catalysis of CRISPR/Cas12a and the G-quadruplex/hemin DNAzyme. In the absence of ATZ, the G-quadruplex/hemin DNAzyme catalyzes oxidation of DA by hydrogen peroxide, leading to the formation of a PDA deposit.

This, in turn, inhibits the photocurrent at the photoanode by blocking the light reaching the electrode and by consuming DA involved in current generation, leading to a measurable photocurrent decrease.

In the presence of ATZ, ATZ can hybridize with an ATZ aptamer (Apt) within an Apt/complementary DNA (cDNA) complex. This hybridization releases the activation strand (cDNA), which activates CRISPR/Cas12a and triggers cleavage of G4. This process leads to a decrease in the amount of the G4/hemin DNAzyme on the electrode, consequently reducing both PDA generation and DA consumption. This disruption of the inhibitory processes ultimately leads to restoration of the photocurrent, providing a quantitative signal for ATZ detection. With a linear photocurrent response to the logarithmic ATZ concentration, the sensor proved effective for determining trace ATZ levels in both environmental and food samples [65].

Shifting our focus from currently used pesticides to legacy persistent organic pollutants (POPs), Miao et al. have turned their attention to the critical issue of dichlorodiphenyltrichloroethane (DDT) detection. Once a widely used agricultural insecticide, DDT and its metabolites are now recognized as POPs. Their alarming stability in nature and resistance to degradation mean they linger in the environment, posing significant toxicological threats to both ecosystems and food safety. They developed an innovative electrochemical impedance sensor that utilizes magnetic Fe_3_O_4_ and PDA MIP nanoparticles (PDA@Fe_3_O_4_-MIP MNPs) for highly sensitive and selective detection of 4,4’-DDT in food samples. The sensor’s core mechanism relied on molecular imprinting, where Bisphenol A (BPA) served as a virtual template to create specific recognition sites for DDT within the PDA layer. Recognition and adsorption of DDT molecules in these recognition cavities led to a measurable increase in the sensor’s electrochemical impedance, allowing for accurate quantification. The as-prepared MNPs could be used not just for specific adsorption but also for efficient extraction of target molecules from food samples due to their magnetism, which allows for easy separation from complex sample solutions. With excellent linearity, a low detection limit, and high selectivity, this method offers a promising and reproducible solution for monitoring DDT contamination [66].

**Figure 5:**
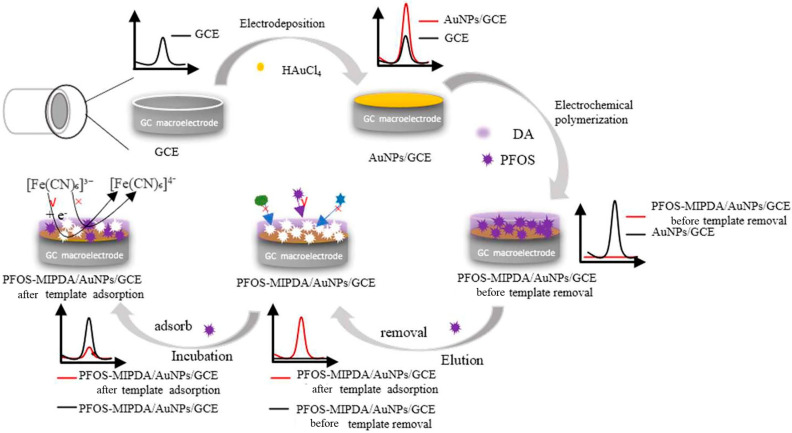
Schematic diagram of preparation of molecularly imprinted PFOS-MIPPDA/AuNPs/GCE sensor and determination of PFOS [68].

Expanding on detection of POPs, another well-known chemical with strong resistance to biological and chemical degradation is Perfluorooctane Sulfonate (PFOS). Unlike the previously mentioned compounds, which were primarily used as pesticides, PFOS was mainly used in a vast array of products and industries due to its unique properties, particularly its ability to repel water, oil, and stains and its exceptional thermal and chemical stability [67]. Because of the widespread existence of PFOS in the environment and its harmful effects on organisms, PFOS was listed in the Stockholm Convention in 2009, restricting its production and use around the world. In 2023, Gao et al. presented an innovative, cost-effective, and rapid electrochemical sensor for highly sensitive and selective detection of trace PFOS. This sensor is based on a GCE produced via electrodeposition with AuNPs and modified with an MIP that is prepared through in situ electropolymerization of DA with PFOS as the template (PFOS-MIPPDA/AuNPs/GCE). PFOS determination was established by DPV using K_3_[Fe(CN)_6_] as the detection probe. When PFOS binds to the recognition sites, it changes the electrochemical properties of the sensor’s surface, affecting the way the probe behaves, and the concentration of PFOS can be determined indirectly. As schematized in Figure 5, the sensor was used to analyze real water samples with satisfactory results, demonstrating the realization of a method characterized by low costs, sensitive detection, and fast operation with potential application prospects for PFOS detection [68].

Beyond pesticides and POPs, another important class of chemical raw materials is phenolic compounds. These are widely used not just in pesticides but also in dyes, cosmetics, secondary colorants, and other products [69]. While these phenols have found widespread use, their lingering presence causes significant environmental damage due to their high toxicity, fire resistance, and water solubility. This imbalance can gravely impact human health, leading to various diseases, including cancer, and even death [70].

Trichlorophenol (TCP) is a phenolic fungicide and herbicide characterized by high stability, bioaccumulation, toxicity, and poor biodegradability. Due to these features, it represents an environmental and biological threat, so it is crucial to have an effective detection method. Selvi et al. opted for an electrochemical method due to its fast detection response, easy sample preparation, good reproducibility, and low instrument cost. In particular, they developed an electrochemical sensor that utilizes a novel α-Bi_2_O_3_ microplate (MP)/PDA-rGO nanocomposite fabricated onto a screen-printed carbon electrode (SPCE). The sensor detects TCP through an irreversible electrochemical oxidation process. The functional groups on PDA-rGO electrochemically interact with TCP. During detection, 2,4,6-trichlorophenol is oxidized to 2,4,6-trichloroquinone. Electrochemical oxidation of TCP generates a measurable current that is correlated with the TCP concentration. This process allows quantification of TCP over a specific range. While other interfering species may be present, the sensor was designed to primarily interact electrochemically with the functional groups of TCP, ensuring that the measured current corresponds specifically to the TCP concentration [71].

Among the tested interferents, we found catechol (CAT), resorcinol (RC), and hydroquinone (HQ), phenolic compounds characterized by structures similar to TCP and environmental co-presence. While Selvi et al. considered these molecules just as interferents, two different research groups focused on detection of these molecules; notably, Fu et al. designed a sensor for RC, while Jiao et al. focused on simultaneous determination of HQ and CAT.

As previously noted, their persistent presence leads to substantial environmental harm; therefore, it is of great significance to design a new sensor for selectively detecting phenolic compounds, especially for determination in real samples. Fu et al. developed a ternary hierarchical porous nanoprobe based on a combination of cuttlefish ink and bimetallic Au@Ag nanoclusters for selective determination of RC. PDA nanoparticles extracted from cuttlefish served as a base for immobilizing Au@Ag core–shell bimetallic nanoclusters (NCs), which were formed through reduction and electrostatic interaction. The PDA-Au@Ag bimetallic NCs were subsequently subjected to a topological transformation (TTF) that converted the compact structure of PDA into a nitrogen-doped porous carbon (NPC) functionalized by the bimetallic Au@Ag. This hierarchical porous nanostructure significantly increased the specific surface area and provided numerous transmission channels, which were crucial for the enhanced sensing performance. The NPC-Au@Ag NCs were then immobilized on the surface of a graphite layer at the center of a Highly Oriented Pyrolytic Graphite (HOPG) electrode. This NPC-Au@Ag-HOPG sensor operates based on irreversible electrochemical oxidation of RC into the corresponding quinone, resulting in a measurable anodic peak current that linearly increases with the RC concentration, providing a quantitative measure. With its biomaterials and bimetallic nanostructures, this sensing platform has shown promise for practical environmental use [70].

As mentioned before, Jiao et al. focused on HQ and CAT instead. These compounds are isomers of dihydroxybenzenes, meaning that they have similar structures and characteristics. This often leads to their coexistence and mutual interference during analytical detection. Consequently, developing highly sensitive methods for their simultaneous determination is crucial. The researchers developed a sensor based on a Mo-, N-, and S-doped interconnected porous carbon sphere material (Mo, N, S-IPCS) for simultaneous determination of HQ and CAT. They used PDA as a template and nitrogen source, thioacetamide (TAA) as a sulfur source, and sodium molybdate as a molybdenum source. Through simple ultrasonic and pyrolysis reactions, uniform, porous nanoparticles with a stable conductive layer were formed. Then the Mo, N, S-IPCS solution was dropped onto the surface of the GCE, constituting the final sensor. CV experiments demonstrated that the Mo, N, S-IPCS/GCE electrode shows two independent and distinct quasi-reversible redox peaks for HQ and CAT. In contrast, an unmodified GCE and a calcined PDA/GCE could not separate these peaks. As the concentration of HQ and CAT increases, the current gradually increases, showing a good linear relationship. This novel sensor, characterized by high sensitivity; a wide detection range; and good reproducibility, selectivity, and stability, has also been successfully applied to real river water samples with acceptable accuracy and recoveries [69].

The pervasive threat of various contaminants, ranging from essential pesticides to persistent organic pollutants and widely used phenolic compounds, highlights the urgent need for advanced detection methods. While crucial for agricultural productivity and industrial applications, the environmental persistence and inherent toxicity of these substances pose significant risks to both ecosystems and human health. The innovative electrochemical and photoelectrochemical sensors explored herein represent a substantial leap forward, with PDA emerging as a particularly versatile and essential component of these groundbreaking developments.

**Table 5:** A summary of the analytical parameters discussed in the papers presented in Section 3.3.

Reference	Analyte	Analyte Concentration Range in Real Samples	LOD	Stability	Linear Response Range	Regulatory Threshold
[64]	Dichlorvos in water	From 0 to 6 ± 0.5 × 10^−3^ mg/L	1.318 × 10^−3^ mg/L	After 3 days, current decreased by 6.84%.	0.007–0.025 mg/L	WHO: 0.005 mg/L (HBV); 0.001 mg/L (drinking water) EPA: 0.0058 μg/L (chronic AWQC); 0.001 mg/L (drinking water)
[65]	ATZ in tomatoes, apples, and Lijiang River water	Tomato: 3.91 × 10^−11^ mol/L	3.47 × 10^−13^ mol/L	After 15 days, photocurrent decreased by <6.42%.	1.00 × 10^–12^–1.00 × 10^–5^ mol/L	WHO: 0.002 mg/L (drinking water) EPA: 9.7 μg/L (CE-LOC); 0.003 mg/L (drinking water)
		Apple: 1.66 × 10^−11^ mol/L				
		Lijiang River water: ND				
[66]	DDT in food samples (radish)	0; 102.32; 0.893; 0.093 µM (spiked additions: 0; 100; 1; 0.01 µM, respectively)	6 × 10^−12^ mol/L	After 4 weeks the sensor retained 97.26% of the Rct value.	1× 10^−11^–1× 10^−3^ mol/L	WHO: 0.001 mg/L (drinking water) EPA: 0.0010 µg/L (chronic AWQC, average over 24 h)
[68]	PFOS in lake, canal, and tap water	Tap water: ND; 0.0102; 0.0208; 0.0393 µmol/L	0.0042 µmol/L	After 1 day, peak current decreased to 41.23%.	0.01–8.00 µmol/L	EPA: 4 ppt (MCL of drinking water)
		Lake water: ND; 0.0100; 0.0208; 0.0416 µmol/L				
		Canal water: ND; 0.0105; 0.0195; 0.0393 µmol/L				
		Spiked additions: 0; 0.01; 0.02; 0.04 µmol/L, respectively				
[71]	TCP in water, soil, and food samples	River water: ND; 0.098; 0.99; 4.90; 9.94; 14.81 µM	0.0042 µM	After 12 days, current decreased by ≈7%.	0.019−190.7 and 212.7−1649 µM	WHO: 0.1 mg/L (drinking water) EPA: MCLG of 0.002 mg/L
		Tap water: ND; 0.094; 0.98; 4.91; 9.97; 14.76 µM				
		Soil: ND; 0.097; 0.98; 4.87; 9.78; 14.80 µM				
		Red wine: ND; 0.098; 0.98; 4.97; 9.86; 14.63 µM				
		Apple juice: ND; 0.098; 0.98; 4.98; 9.87; 14.80 µM				
		Spiked additions: 0; 0.1; 1.0; 5.0; 10.0; 15.0 µM, respectively				
[70]	RC in seawater and lake water	ND	0.06 mM	After 36 days, current decreased by 2.01%.	1–100 μM 1.2–4 mM	ND
[69]	HQ in river water	49.81± 2.24 μM and 100.28± 5.13 μM (spiked additions: 50 and 100 μM, respectively)	0.047 μM	After 1 month, the response current was 93.01% of the original response current.	5.0 × 10^−6^ to 1.0 × 10^−2^ M	ND
	CAT in river water	47.21 ± 5.13 and 99.61 ± 3.78 (spiked additions: 50 and 100 μM, respectively)	0.018 μM	After 1 month, the response current was 96.35% of the original response current.	5.0 × 10^−8^ to 2.0 × 10^−3^ M	ND

**Table 6:** A summary of the structural features of the systems discussed in papers presented in Section 3.3.

Reference	Base Electrode	Electrode Modifier	Precursors	Mechanism of Modification
[64]	GCE sensor	ZrO2@PDA	Zirconium chloride octahydrate; dopamine hydrochloride	ND
[65]	FTO electrode	GLD/Cs/ Zn_0.5_Cd_0.5_S/Ti_3_C_2_/G4/hemin DNAzyme	Glutaraldehyde; chitosan; zinc acetate; cadmium acetate dihydrate; dopamine; thioacetamide; hemin; LbCas12a; ssDNA-FQ; crRNA; G-quadruplex, ATZ aptamer, and activator strand DNA	Drop coating method
[66]	GCE sensor	PDA@Fe_3_O_4_-MIP MNPs	Fe_3_O_4_ nanoparticles; dopamine hydrochloride; BPA	Drop casting
[68]	GCE sensor	PFOS-MIPPDA/AuNPs	HAuCl_4_ × 4H_2_O; KCl; dopamine hydrochloride; PFOS	Solution immersion and electropolymerization
[71]	SPCE sensor	α-Bi_2_O_3_ MPs/PDA-RGO	Bismuth nitrate; hydroxylamine hydrochloride; GO; dopamine	Drop casting
[70]	HOPG sensor	NPC-Au@Ag	Gold (III) chloride trihydrate; silver nitrate; cuttlefish bought from a local supermarket	Topological transformation technology and drop casting
[69]	GCE sensor	Mo, N, S-IPCS	Sodium molybdate; thioacetamide; dopamine hydrochloride	Drop casting

## 4. Food Applications

Rapid and selective detection of food contaminants, such as pathogenic bacteria or antibiotic residues, is essential to ensure consumer safety. Traditional techniques are accurate, but they are often slow, expensive, and unsuitable for direct analysis of samples. Electrochemical sensors represent a promising alternative because of their speed, sensitivity, and ease of use. The examples discussed in this section are summarized in Table 7 and Table 8.

**Figure 6:**
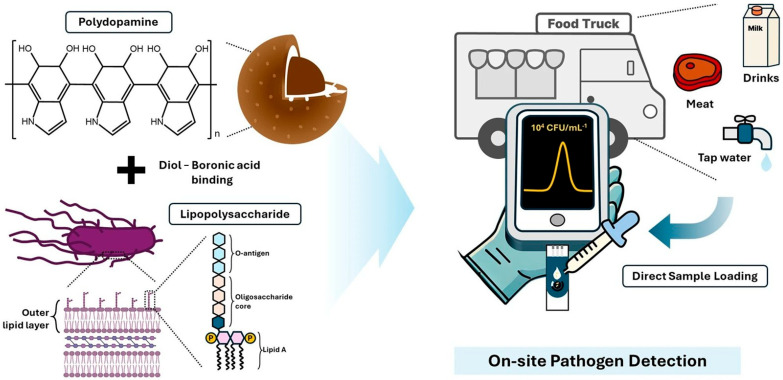
Schematic of MIP preparation and application for on-site pathogen detection in food samples [72].

The bacterium *Salmonella typhimurium* (*S. typhimurium*), a frequent contaminant of food products, is a major cause of gastrointestinal infections, with symptoms such as diarrhea, fever, and abdominal cramps. This disease is particularly dangerous for the most vulnerable populations, highlighting the need for rapid and reliable analytical methods for in situ detection. To this end, Lee et al. developed an electrochemical sensor based on an MIP for selective detection of *S. typhimurium*, as schematized in Figure 6. Their strategy involves using only the lipopolysaccharide (LPS) of *S. typhimurium* as a mold for molecular imprinting, rather than the entire bacterium. This approach simplifies the production of MIPs and reduces the risks associated with handling live bacteria while maintaining high molecular specificity. PDA was selected as the imprinting material due to its unique surface adhesion properties and low cost. MIP synthesis involves the formation of PDA nanoparticles that are selectively bound to LPS, then coated with a second layer of PDA to form a shell structure. Subsequent removal of the LPS template results in MIPs with selective recognition cavities that complement the target analyte. The sensor was fabricated by depositing MIP on a carbon working electrode, and DPV measurements were used to detect the presence of *S. typhimurium*.

The developed method allowed direct detection of whole bacteria, as confirmed by analysis of inoculated samples of real food (tap water, milk, and pork), without the need for pretreatment. The system showed a low detection limit; a wide linear range; and high sensitivity, reliability, and specificity [72].

**Table 7:** A summary of the analytical parameters discussed in papers presented in Section 4.

Ref	Analyte	Analyte Concentration in Real Samples	LOD	Stability	Linear Response Range	Regulatory Threshold in Potable Water	Regulatory Threshold in Superficial Water
[72]	*Salmonella typhimurium* (*S. Typhimurium*) in food	Tap water, milk, and pork ≈ 10^4^–10^6^ CFU/mL (spiked additions)	10 CFU/mL	ND	10^2^–10^8^ CFU/mL	ND	ND
[48]	Chloramphenicol in dairy products	Pure milk: 0.47; 0.98; 5.04 × 10^−5^ mol L^−1^	2.0 × 10^−8^ mol L^−1^	After 30 days, the current response was 90.5% of the initial value.	5.0 × 10^−7^–5.0 × 10^−4^ mol L^−1^	ND	ND
		Milk powder: 0.46; 0.97; 5.08 × 10^−5^ mol L^− 1^					
		Yoghurt: 0.45; 0.99; 5.03 × 10^−5^ mol L^−1^					
		Spiked additions: 0.50; 1.00; 5.00 × 10^−5^ mol L^−1^, respectively					

**Table 8:** A summary of the structural features of the systems discussed in papers presented in Section 4.

Reference	Base Electrode	Electrode Modifier	Precursors	Mechanism of Modification
[72]	Carbon working electrode	LPS, DA	LPS-imprinted MIP	Drop casting
[48]	GCE	DA, straw biogas residue, β-cyclodextrin	Biogas residue biochar @polydopamine (BRB@PDA), β-cyclodextrin	Drop casting and electrodeposition

Regarding antibiotic residues, chloramphenicol (CAP) is a widely used antibiotic in the medical and veterinary fields due to its low cost and broad-spectrum activity. Traces of CAP have been detected in dairy products, making it possible to accumulate this compound in the body through food, with potential serious effects on human health, including aplastic anemia, leukemia, and gray baby syndrome. In this context, Wang et al. developed an electrochemical sensor based on biochar derived from corn straw for detection of CAP in dairy products. Specifically, the biochar was coated with PDA, which was chosen for its excellent adhesive and CAP attraction properties, and deposited on a glassy carbon electrode (GCE) to improve its electronic conductivity. β-Cyclodextrin (β-CD), which is capable of forming specific hydrogen bonds with the target molecule, was electrodeposited on the surface of the BRB@PDA/GCE to increase its selectivity towards CAP. The resulting sensor was used for electrochemical detection of CAP by CV and SWV in real samples such as milk, yogurt, and milk powder, through experiments with standard additions. The sensor demonstrated a wide detection range and a low detection limit, as well as high reproducibility, stability, and specificity, representing a sustainable and cost-effective alternative to traditional nitrogen-doped carbon electrodes [48].

## 5. Others

As we have seen in previous paragraphs, electrochemical sensors are increasingly favored for pollutant detection; they offer straightforward operation, rapid responses, low energy use, enhanced sensitivity, and in situ analysis. In sensor construction, choosing substrate materials with excellent conductivity and selecting recognition components with strong binding abilities are key to efficiently converting the interaction between the analyte and the sensor into accurate and reliable electrochemical signals. Many functional nanomaterials, such as gold nanoparticles, MXene materials, MOF materials, carbon materials, and conductive polymers have been used for sensors. However, some researchers focused on developing alternative sensors or improving existing ones by leveraging PDA to enhance adhesion, avoid oxidative reactions, and increase surface hydrophilicity, as well as for in situ NP formation. The examples discussed in this section are summarized in Table 9 and Table 10.

Gonzalez-Martinez et al. focused on the use of paper as an alternative when creating disposable electrochemical sensing devices. The first challenge consisted of making the paper conductive, and this was achieved by gold electroless deposition onto prestressed polystyrene (PS) substrates, where the substrates were first coated with a PDA layer to enhance metal adhesion. The electrodes fabricated using this method possessed an intrinsic roughness that endowed them with a larger surface area than electrodes fabricated through sputtering deposition. These properties make them attractive for two applications: as electrochemical devices and as surface-enhanced Raman scattering (SERS)-based sensors. The performance of the paper-based electrodes was tested by detecting mercury ions in aqueous solutions. Hg^2+^ in solution was reduced to solid mercury on the surfaces of the electrodes and was then re-oxidized into Hg^2+^ ions, releasing electrons in the process. This flow of electrons was measured as a linear increase in the current with an increase in the concentration. The second application, as surface-enhanced Raman scattering (SERS) sensors, was tested by tuning the surface roughness of the gold coating for detection of thiophenol. In both cases, they reached an acceptable limit of quantification and developed robust conductive papers that could also be suitable for other applications, including energy storage, catalysis, and flexible electronics [73].

Jiang et al. instead addressed the limit of a silicon (Si) substrate as a photoelectrode in light-addressable electrochemistry (LAE). Indeed, Si is easily oxidized, resulting in the formation of a silicon oxide (SiOx) layer that is electronically insulated and blocks charge transfer between the electrode and the electrolyte. Through deposition of a thin layer of PDA film on the Si substrate and subsequent carbonization, they were able to prevent the natural oxidation of Si. Moreover, the carbonized PDA (cPDA) layer could prevent anodic oxidation when applied as an electrode. The cPDA layer-modified Si substrate showed excellent photoelectrochemical performance and stability, maintaining its signal without any significant decrease [22].

Qi et al. instead focused on improving polymeric membrane ion-selective electrodes (ISEs), particularly addressing biofouling, which is the undesirable attachment and development of microorganisms into biofilms on sensor surfaces. They demonstrated that surface modification effectively enhanced the anti-biofouling properties of these ISEs. Their approach combined anti-adhesive and antimicrobial materials for excellent biofouling resistance. The ISE membrane was modified with hydrophilic PDA, followed by in situ formation of AgNPs. This was achieved by coating the ISE surface with a PDA layer through dopamine’s self-polymerization, which then reduced silver ions to Ag, allowing synthesis of AgNPs in situ without an additional reducing agent. The PDA modification boosted the membrane’s anti-adhesive properties by increasing its surface hydrophilicity, while the AgNPs provided strong antimicrobial properties. They proved that the AgNP-coated K+-ISE effectively resists biofouling, showing both low bacterial viability and low adhesion, all while maintaining its original potentiometric ion response [74].

These studies highlighted that PDA is proving to be a transformative material for enhancing existing sensors and enabling new designs. Specifically, PDA’s unique properties, such as its strong adhesion, excellent biocompatibility, and rich functional groups, have been ingeniously leveraged to enhance sensor performance. From enabling conductive paper-based sensors for heavy metals and organic compounds to preventing silicon photoelectrode degradation and imparting excellent anti-biofouling properties to ISEs, PDA is a crucial component. Its unique characteristics are key to developing more robust, stable, and high-performing electrochemical sensors for diverse environmental and analytical applications.

**Table 9:** A summary of the analytical parameters discussed in papers presented in Section 5.

Reference	Analyte	LOD	Stability	Linear Response Range	Regulatory Threshold
[73]	Hg^2+^ in solution	0.3 ppb	ND	1 to 10 nM	WHO: 0.006 mg/L (drinking water) EPA: 0.002 mg/L (drinking water)
	Thiophenol	3 ppb		1 to 20 μM	NA
[22]	Ascorbic acid; HQ	NA	After 32 h, photocurrent decreased by 20%.	NA	NA
[74]	K^+^	10^−5.4^ M	ND	10^−5^ to 10^−2^ M	NA

**Table 10:** A summary of the structural features of the systems discussed in papers presented in Section 5.

Reference	Electrode Substrate Type	Electrode Modifier	Precursors	Mechanism of Modification
[73]	Paper-based electrode	AuNP PDA	H[AuCl_4_]; dopamine hydrochloride	Electroless deposition
[22]	n-type Si electrode	cPDA	Dopamine	Solution immersion and annealing
[74]	GCE (K+-ISE)	PDA-AgNPs	Dopamine; silver nitrate	Solution immersion

## 6. Outlook and Perspectives

Melanin-related materials have found applications in fields with relevant social and economic impacts, including environmental monitoring with electrochemical sensors. Nevertheless, in our opinion, the potentialities of these materials in this context have not been completely developed yet.

Notably, nearly all of the recently developed electrochemical sensors discussed in this review are based on PDA derived from oxidative polymerization of DA. These sensors take advantage of PDA’s strong adhesion, multifunctionality, and anticipated biocompatibility as a natural eumelanin mimic. Thanks especially to its multifunctionality, PDA is mostly used in electrochemical sensors to enhance their sensitivity, while the issue of conductivity is, in most cases, ignored. Therefore, possible developments in the application of melanin-related materials in electrochemical sensors could involve the following: 

*Alternative melanin-related materials*. PDA is surely a material with incredible properties. Nevertheless, other forms of biomimetic melanin are attracting increasing attention.

One example is allomelanin, which results from oxidative polymerization of 1,8 di-hydroxynaphthalene. The electrochemical properties of this kind of material are still widely unexplored, especially in the form of thin films (nanometric thickness). Although the chemistry of the precursor is, in part, different from DA, it may be processed in a similar way to DA to give composites with new properties. From the point of view of biocompatibility, we would like to stress that allomelanin is the typical form of melanin present in fungi. Although this material has not yet reached the same popularity as PDA, it has been demonstrated to present superior features with respect to PDA for several applications [41,75]. Hence, its integration in electrochemical sensors should be developed. Copolymerization of DA with other precursors has also been only marginally developed.

*Controlling conductivity*. The actual conductivity of melanin when introduced in electrochemical sensors is poorly controlled. Although melanin, mostly in the form of PDA, is typically used to enhance the interactions between the electrode and the target analyte and hence increase the sensitivity, the effect on electrode conductivity is often ignored. The deposition/incorporation conditions are, for this purpose, very important.

*Alternative electroanalytical methods.* The most efficient and recent examples of melanin-based electrochemical sensors were designed by exploiting conventional CV, DPV, and SWV. Nowadays, new and more sophisticated electroanalytical techniques are available, like EIS (Electrochemical Impedance Spectroscopy) and PEC (Photoelectrochemistry), which can also be exploited to design microfluidic devices. These advanced techniques were recently used to characterize melanin [76] and may be used in the future to design advanced melanin-based electrochemical sensors.

*Improving stability*. The mechanical stability of PDA films can be critical. It affects the long-term activity of electrochemical sensors. Moreover, PDA films and melanin-related materials, in general, are known to be affected by oxidation/reduction. Their original properties can change, or they can even undergo degradation and dissolution. Long-term stability may be important when using electrochemical sensors for environmental applications, and it should be investigated in detail.

*Graphitization*. Several methods have been proposed to modify, totally or in part, the electrochemical properties of PDA. Some of these methods require exposure to high temperatures in the absence of oxygen, and they may be detrimental to sensor electrodes; nevertheless, their possible effects should be analyzed. Other treatments based on focused laser light are expected to be less aggressive and could be applied locally and in a better-controlled way. Using this kind of treatment, the responses of melanin-based electrochemical sensors may be improved, controlling, in particular, the conductivity of the sensor electrodes.

A few recent reviews have been published about the application of melanin-related materials for electrochemical detection of pollutants in the environment and food [29,77]. The main differences with respect to the present work are reported in Table 11.

**Table 11:** Recent reviews about melanin-based electrochemical sensors for the environment and food.

Reviews	Application	Topic of Interest	Year of Publication
This review	Environmental and food	Assesses the problem of melanin’s low conductivity and proposes innovative methodologies to increase it.	2025
Recent Advances in Polydopamine-based Electrochemical Biosensors	Diagnostic and therapeutic	PDA is only described as able to increase the electrocatalytic properties of an electrode, without focusing on its limitations.	2022
Polydopamine films: Electrochemical growth and sensing applications	General/environmental	Focuses on the influence of other parameters, such as thickness, on PDA’s conductivity.	2022

## 7. Conclusions

Despite their moderate conductivity, melanin-related materials, in particular PDA, are increasingly being applied in the field of electrochemical monitoring of the environment. The main strengths of these materials are their high biocompatibility and their high density of functional groups, which allow them to enhance the sensitivity of electrochemical sensors. Additionally, PDA has been reported to be able to attach to any kind of surface, and its formation, resulting from oxidative polymerization of DA, can be easily achieved electrochemically. Coating and functionalization of pre-existing electrodes is hence very simple and promising. Most recent applications of melanin-related materials, in particular PDA, in electrochemical sensing of environmentally significant targets demonstrate that enhanced sensitivity can be achieved. We believe that the full potential of this analytical approach has not been completely exploited and that new design strategies will lead to the production of even more effective electrochemical sensors.

## Data Availability

No new data were created or analyzed in this study.
